# “Caminando Con Riesgo”: perceptions of occupational injury, workplace safety and workers rights among Spanish-speaking hospitalized patients

**DOI:** 10.3389/fpubh.2024.1347534

**Published:** 2024-04-23

**Authors:** Amy Zeidan, Juliana Cortes, Hannah Marcovitch, Roxana Chicas, Randi N. Smith, Alessandra Stevens, Elizabeth Zambrana, Shelly Anand

**Affiliations:** ^1^Department of Emergency Medicine, Emory University School of Medicine, Atlanta, GA, United States; ^2^Nell Hodgson Woodruff School of Nursing, Emory University, Atlanta, GA, United States; ^3^Emory University School of Medicine, Atlanta, GA, United States; ^4^Department of Surgery, Emory University School of Medicine, Atlanta, GA, United States; ^5^Sur Legal Collaborative, Atlanta, GA,United States

**Keywords:** occupational injury, occupational health, workers rights, immigration, Occupational Safety and Health Administration

## Abstract

**Introduction:**

Occupational health disparities are well documented among immigrant populations and occupational injury remains a high cause of morbidity and mortality among immigrant populations. There are several factors that contribute to the high prevalence of work-related injury among this population and those without legal status are more likely to experience abusive labor practices that can lead to injury. While the work-related injuries and experiences of Spanish-speaking workers have been explored previously, there is a paucity of literature documenting injury among hospitalized patients. Additionally, there are few documented hospital-based occupational injury prevention programs *and* no programs that implement workers rights information. The purpose of this study was to further explore the context of work related injuries primarily experienced by Spanish speaking patients and knowledge of their rights in the workplace.

**Methods:**

This was a semi-structured qualitative interview study with Spanish speaking patients admitted to the hospital for work related injuries. The study team member conducting interviews was bilingual and trained in qualitative methodology. An interview guide was utilized for all interviews and was developed with an immigrant workers rights organization and study team expertise, and factors documented in the literature. Participants were asked about the type and context of the injury sustained, access and perceptions of workplace safety, and knowledge of participants rights as workers. All interviews were conducted in Spanish, recorded, transcribed in Spanish and then translated into English. A codebook was developed and refined iteratively and two independent coders coded all English transcripts using Dedoose. Interviews were conducted until thematic saturation was reached and data was analyzed using a thematic analysis approach.

**Results:**

A total of eight interviews were completed. All participants reported working in hazardous conditions that resulted in an injury. Participants expressed a relative acceptance that their workplace environment was dangerous and acknowledged that injuries were common, essentially normalizing the risk of injury. There were varying reports of access to and utilization of safety information and equipment and employer engagement in safety was perceived as a facilitator to safety. Most participants did have some familiarity with Occupational Safety and Health Administration (OSHA) inspections but were not as familiar with OSHA procedures and their rights as workers.

**Discussion:**

We identified several themes related to workplace injury among Spanish speaking patients, many of which raise concerns about access to workplace safety, re-injury and long-term recovery. The context around immigration is particularly important to consider and may lead to unique risk factors for injury, recovery, and re-injury both in the workplace and beyond the workplace, suggesting that perhaps immigration status alone may serve as a predisposition to injury. Thus, it is critical to understand the context around work related injuries in this population considering the tremendous impact of employment on one’s health and financial stability. Further research on this topic is warranted, specifically the exploration of multiple intersecting layers of exposure to injury among immigrant populations. Future work should focus on hospital-based strategies for injury prevention and know your rights education tailored to Spanish speaking populations.

## Introduction

It has been suggested that immigration status alone *is* a social determinant of health (1). Indeed, immigration status impacts all areas of an individual’s life, including access to safe and healthy work environments (2). Occupational health disparities are more prevalent among immigrant populations who are at an increased risk of workplace morbidity and mortality (3). Immigrant populations, especially those without legal status, are more likely to work in physically demanding jobs that are hazardous, such as construction, agriculture, maintenance, and service occupations that have high incidence rates of nonfatal occupational injuries and illnesses (4, 5). Additionally, the workplace fatality rate is nearly 50% higher for Latino workers than their non-Latino counterparts (6).

As immigrant populations in the United States continue to expand, they will likely account for an increasing percentage of those working under high risk labor conditions. In the state of Georgia, one in eight workers is an immigrant, primarily concentrated in sectors with hazardous working conditions (e.g., manufacturing, construction) (7). Similarly, a retrospective review conducted at a high-volume Emergency Department in Atlanta revealed that of 267 non-English-speaking individuals hospitalized for an injury, nearly 25% were hospitalized for a work-related injury; 95% of whom were Spanish speaking (8). The high rate of work-related injuries among immigrant populations is likely multifactorial, including but not limited to language and communication barriers, lack of immigration status, discrimination and structural barriers to labor protections, training, provision, and use of safety equipment (4, 9). Those without legal status are more likely to be subject to predatory and exploitative labor practices due to fears of reports to immigration enforcement (10). Relatedly, immigrant populations make up a larger percentage of labor trafficking survivors, with the agricultural industry being the largest perpetrator, followed by domestic work and construction (11). It is important to note that immigrants are protected under federal laws. Occupational Safety and Health Administration (OSHA) protections apply to all workers, regardless of their legal status (12). Some states have ‘state plans’ that add additional protections to workers but that is note the case for the state in which this study occurred (Georgia) (13). Employers are thus responsible for following federal OSHA standards (12). OSHA has clearly defined worker rights and protections, noting that under OSHA standards, employees have the right to a safe and healthy work environment and protections from retaliation if advocating for this (14). This includes the right to file a complaint against an employer and be protected from relation via ‘whistleblower’ protections. The challenge is that not all of these protections are enforced and state laws protecting immigrant workers vary such that in some states, an individual without legal status may still face risks of ‘discovery’ by immigration enforcement if attempting to pursue a case (4). Accountability, enforcement, and weak penalties have also been cited as challenges at OSHA. The current OSHA budget does not appropriately cover staffing to process and enforce regulations and there are concerns about their actual ability to protect workers from retaliation (15). Despite existing initiatives that attempt to reach immigrant workers, including the development of Spanish-Language Compliance Assistance Resources and dedicated ‘Hispanic Outreach’ tools (training resources, compliance programs, etc.), immigrant workers remain exceptionally vulnerable and experience a disproportionate amount of work related injuries (16–18).

Experiences of safety and injury among Spanish-speaking workers has been studied previously however few studies explore perspectives of hospitalized patients. Prior studies have resulted in important interventions; however, despite the frequency with which patients present to the hospital for work related injuries, hospital-based interventions are lacking (19, 20). Additionally, interventions largely focus solely on injury prevention rather than emphasizing both injury prevention *and* Know Your Rights (KYR) training. Hospital settings may represent an important and understudied point of intervention for injury prevention and KYR programming. In collaboration with a local community organization, Sur Legal Collaborative (Sur Legal), we designed a study to characterize the contextual factors around work-related injuries requiring hospitalization, as well as knowledge of OSHA and workers rights among Spanish-speaking patients admitted to the hospital for a work related injury.

## Methods

### Study design

This was a qualitative semistructured interview study with Spanish-speaking individuals admitted to the hospital for a work-related injury between June and August 2023. This method was chosen as a means of exploring contextual factors within the work environment that led to injury and hospitalization, barriers and facilitators to workplace safety, and knowledge of the OSHA and workers rights.

### Study setting and population

Participants were eligible for this study if they were adults (≥18 years of age) admitted to the hospital for a work related injury and were Spanish speaking, including those who were bilingual. Patients were excluded if they were unable to provide consent. This study was conducted at a large, academic, public hospital in Georgia; this center is the only level 1 trauma and emergency care center in Atlanta. The hospital is a public safety-net hospital serving a largely un-and underinsured population, with over 140,000 ED visits and 7,500 trauma activations annually. According to the hospital’s language interpretive services department, 13% of patients receiving care at the hospital have a non-English Language Preference (NELP), the majority of whom speak Spanish (7%). The study was reviewed and approved by the University’s Institutional Review Board and complies with the consolidated criteria for reporting qualitative studies (COREQ).

### Study protocol

Participants were approached in person by a bilingual research assistant in the Emergency Department or inpatient Trauma service. The research assistant screened patients admitted to the trauma service via the Electronic Medical Record to identify their preferred language and mechanism of injury. Participants were approached in their private hospital room, consented, and all interviews occurred in the participant’s room. Participants were selected purposively to include those with Spanish as their preferred language and who were admitted for a variety of work related injuries. All interviews were conducted using an interview guide which was developed, piloted and refined by the study team. The study team has content expertise in occupational health, environmental health, nursing, public health, emergency medicine, trauma surgery and critical care, injury prevention, labor law, and workers rights. Questions were developed to explore contextual factors related to the workplace environment, safety procedures, and knowledge of OSHA and workers rights. Participant demographics were collected at the end of the interview and included gender, race/ethnicity, age, country of birth, years living in the United States, preferred language, other languages spoken, number of years in current job, highest education level completed, and whether they had health insurance. We did not ask about immigration status as this question may have been a barrier to establishing trust and/or discouraged workers from participating in this study.

Interviews were conducted in-person with a bilingual study team member with qualitative interviewing experience (JC) who had recently completed her masters in public health. The interviewer had no previous interaction with study participants, although she did have experience enrolling patients at the study site for trauma specific studies (none of which included the participants from this study). All interviews were recorded, professionally transcribed and translated, and conducted until thematic saturation. Thematic saturation is generally reached at 6 to 12 interviews (21). All participants provided verbal consent at the beginning of the interview and were informed that they could terminate at any time, although no participants did so. Interviews lasted approximately 45–60 min. Three additional individuals were approached but the study team ultimately determined they were not appropriate for inclusion given persistent confusion (related to their injury). Participants were provided language concordant Know Your Rights Information developed by the Sur Legal Collaborative if desired.

### Data analysis

We adopted a thematic analysis approach to analyze all qualitative data, as detailed by Braun and Clarke (22). Following review of the English transcripts, study team members with experience in qualitative analysis developed an initial codebook and coded a sample of transcripts (AZ, JC, HM). The same study team members met throughout the coding process to refine and finalize codes and all coding differences were resolved by consensus. All transcripts were coded independently by two team members (JC and HM) using Dedoose, a qualitative coding software. Themes were derived using a semantic approach, whereby patterns are identified explicitly, but latent concepts were also assessed in order to explore foundational ideas. Given the potential for inherent beliefs and biases that can influence interpretation, the study team prioritized reflexivity to ensure these did not impact data analysis.

### Community partnership

An important component of this study was the development of all study components in partnership with Sur Legal. Founded in October 2020, Sur Legal is a woman of color-run and led legal nonprofit organization based in Georgia working at the nexus of labor rights, immigrant rights, and mass decarceration. Sur Legal Collaborative was founded in response to COVID-19 by a career trial attorney with the US Department of Labor who witnessed immigrant workers and low income workers of color being designated as essential workers but knowing nothing about their labor rights, particularly under the OSHA. The mission of the organization is to democratize legal knowledge so that immigrant and working class communities are empowered with the tools they need to hold abusive employers accountable. Sur Legal contributions were invaluable in the development of the study design, interview guide, and interpretation of findings.

## Results

A total of eight interviews were completed with seven men and one woman. Participants self-identified as Latino or Hispanic and were from Mexico (*n* = 3), Guatemala (*n* = 3), Honduras (*n* = 1) and El Salvador (*n* = 1). The mean age was 39 with a range of 23–69 years old. The mean number of years living in the U.S. was 14 with a range of 3–30 years. All participants reported Spanish as their preferred language and two reported also speaking an indigenous language. They had worked in their current job for a range of 3 months to 8 years with a mean of 3.3 years. The highest level of education completed varied from 10 years of age (primary school) to 1 year of college with 14 years old being the mean age of highest education completed. Most did not have insurance (*n* = 6). See Table 1 for Themes and Representative Quotes.

**Table 1:** Themes and representative quotes.

Theme	Representative quote
Hazardous Workplace Conditions	*Any tool, no matter how small and harmless it may seem, is dangerous and can cause damage, perhaps minimal, but it will cause damage […] Construction is not just about coming in and saying, you know what, I’m going to start working. No, you have to know what you are going to do during the day, plan yourself as a person and what you are going to need as tools. All construction is dangerous. Everything is dangerous. 50–55 year old Man, Landscaper Being alone and on top of a ladder, at any moment it can move. It’s not like when you have someone holding it or something like that. 40–45 year old Man, Construction Worker*
Acceptance of Hazardous Workplace Conditions	*Well, I walk with risk […] all the time we walk with unsureness because we work with heights. 20–25 year old man, Roofer Because I do not have any papers. So, since we immigrants are here in the United States, we come to work whatever we can get. We work in construction, all that. That’s where we get the most work, like us. 25–30 year old Man, Torch Cutter*
Perception of Recovery and Return to Work	*But if you lower your morale and become negative, I do not see how anyone is going to have a success or a good result. I have to be strong and get ahead, because I have obligations in life. 50–55 year old Man, Landscaper One is out of fear. Fear and nothing more. Second, because I’m not going to withstand anymore. It has affected me because I am not doing my normal functions as I have always done. –29 year old Man, Torch Cutter Because now I see it’s already dangerous. I do not want to this to happen again. 20–25 year old Man, Roofer*
Workplace Safety	*We never talk about safety. We just go and talk about work. 40–45 year old Man, Construction Worker That’s why you do not use security, because you trust that it’s low, nothing happens to me. 25–30 year old Man, Construction Worker*
Employer Engagement	*There is a good relationship between employer and workers. There are talks about work, how to take care of ourselves, how to protect ourselves and how to use the work tools and protections to take care of our bodies. 50–55 year old Man, Landscaper*
Knowledge of OSHA and Workers Rights	*I used to work in building construction and there, yes, they require you to wear boots, helmet, glasses, gloves and they are always saying if you look at them without a helmet: “Put on your helmet, if OSHA comes, they will give us a ticket.” 25–30 year old Man, Construction Worker These people never talk to you about rights, they talk to you about work, just that you have to work and arrive early or arrive at the time you have to arrive. But they do not tell you: “If you hit this right” or, “We are going to give you this, if this happens to you” no, almost never. I do not think any employer almost offers you rights, most employers never offer you anything almost. 40–45 year old Man, Construction Worker*

Participants interviewed describe their work as the following: construction (framing the exterior of houses, installing sheetrock), ironing clothes (using a hand iron to iron clothes), landscaping/hardscaping (demolishing existing outdoor structures and building outdoor kitchens and decorative walls), sandblasting (cleaning/smoothing hard surfaces with a dedicated machine), painting (painting both inside and outside surfaces of residential houses/apartments), torchcutting (cutting metal materials with fire using a torch, roofing removing the existing roof and/or siding of residential buildings and replacing with new roof/siding). The mechanisms of injuries were highly variable and reported as the following: the participant working in landscaping/hardscaping noted his foot was run over by a large machine used to transport heavy materials; the participant working in sandblasting noted he was on a ladder pouring sand when he was struck by the material causing him to fall off a 10 foot ladder; the participant working as a torchbearer was attempting to remove aluminum that was stuck in his machine when it exploded because of the heat; the participant working as a painter reported falling from a ladder that became unsteady while he was painting; the participant working in construction noted he was on a ladder replacing sheetrock inside a house when the ladder became unstable and moved, causing him to fall; the participant who irons clothes described a steam burn from ironing; one of the participants working as a roofer describes falling from a room because the rope for his harness ran out while the other participant working as a roofer experienced a similar problem when the rope holding him to his harness slipped out from where it was tied, causing him to fall to the ground.

### Workplace conditions

#### Hazardous workplace conditions

All participants described their work environment as hazardous or ‘risky.’ Many work with equipment that can cause serious injury if the equipment were to malfunction or if an accident were to happen and/or an environment where an accident could potentially be lethal (e.g., working at heights). One participant highlights this risk:

*Being alone and on top of a ladder, at any moment it can move. It’s not like when you have someone holding it or something like that.*

#### Acceptance of hazardous workplace conditions

When describing their work environments, respondents conveyed a general *acceptance* of working in hazardous conditions. They regularly commented on the constant risk at work (e.g., working in extreme weather, at heights, with heavy machinery), saying that ‘this is just what happens’ when describing work related injuries. Some noted that they felt they had few other options in terms of access to work:

*Because I don’t have any papers. So, since we immigrants are here in the United States, we come to work whatever we can get. We work in construction, all that. That's where we get the most work, like us.*

For most participants, the current injury was their first serious injury; however, many commented on several instances where they had minor injuries in which they did not seek medical care (e.g., cuts, falls, burns), acknowledging that minor injuries are common in their environment. Most respondents noted that their injury was not anticipated but was also not surprising given the nature of their high risk work environments. Most injuries were a result of an accident or equipment malfunction, generally perceived to be a known risk of their job:

*Well, I walk with risk […] all the time we walk with unsureness because we work with heights.*

Several also commented on observing co-workers experience both major and minor injuries.

### Perceptions of recovery and return to work

When describing their current injury and anticipated recovery, participants were seemingly pragmatic about their injury and recovery. They often responded with statements about the factual components of their recovery as told to them by their medical teams (e.g., surgical plan, recovery timeline) and/or responded with forward-focusing comments; the need to be optimistic or ‘strong’ in order to get through their recovery. One participant describes this sentiment:

*But if you lower your morale and become negative, I don’t see how anyone is going to have a success or a good result. I have to be strong and get ahead, because I have obligations in life.*

Participants described a range of feelings about returning to work, some noting they had not thought about their return, others expressing the desire to return quickly, and others describing the inability to return to work (due to fear or physical challenges). One participant describes fear of returning to a dangerous environment:

*Because now I see it’s already dangerous. I do not want to this to happen again.*

The desire to return to work was most often out of necessity. Some expressed interest in returning to work because of the unique and highly specialized skills they had related to their occupation (describing it as an ‘art’):

*I will try because I like this job. For me it is an art. Very well, yes, and I have no other type of work in mind, but I am going to try.*

### Workplace culture

#### Workplace safety

Despite working in hazardous conditions, there were varying levels of workplace safety precautions described. Some participants commented that there were almost no precautions at work, while others noted they had daily safety checks and were sent home if they did not have the right safety equipment. One participant describes the lack of safety precautions at work:

We never talk about safety. We just go and talk about work.

Participants described several processes they and/or their coworkers follow to ensure safety at work but these were not necessarily employer driven. There were also varying levels of safety equipment used, some provided by employers and others that employees were required to provide. Many described instances in which they opted not to use some of their safety equipment because it was uncomfortable or made their job more difficult.

#### Employer engagement

Participants described employer engagement as a facilitator to ensuring a safe environment. Feeling safe at work was often related to whether their employer prioritized safety. Employees perceived safer work environments if their employer emphasized safety as part of their workplace culture.

*There is a good relationship between employer and workers. There are talks about work, how to take care of ourselves, how to protect ourselves and how to use the work tools and protections to take care of our bodies.*

On the contrary, participants expressed more concern about their safety if their employer was absent and/or did not prioritize safety precautions.

#### Knowledge of OSHA and workers rights

When asked about OSHA, participants had some awareness of OSHA as a monitoring organization but had limited knowledge of OSHA standards or requirements. Similarly, they had limited knowledge of their rights and could not comment on any of their rights as workers. Most participants had not received any information about OSHA or their rights as workers and most had very limited access to safety information or training. One participant highlights this:

*These people never talk to you about rights, they talk to you about work, just that you have to work and arrive early or arrive at the time you have to arrive. But they don’t tell you: “If you hit this right” or, “We are going to give you this, if this happens to you” no, almost never. I don’t think any employer almost offers you rights, most employers never offer you anything almost.*

Notably, respondents described limited formal training in general, noting that informal or ‘on the job’ training was the norm with varying levels of safety training incorporated into their formal or informal training. Most participants felt there could be more precautions in place to make their workplace safer.

#### Participant driven suggestions

Participants were asked for suggestions about improving workplace safety and commented on several potential opportunities including a brochure or booklet about safety and their rights as workers, a website or organization that provided safety information, a class or video on how to avoid accidents, short daily trainings on how to avoid accidents, and more safety training in general. They commented on the desire for their employer to be more invested in safety and to provide necessary safety equipment and support for workplace safety.

## Discussion

In this study, Spanish-speaking patients hospitalized for a work related injury acknowledged and essentially normalized the constant risks they face at work. The level of workplace safety procedures varied from strict daily procedures to no precautions and employer engagement in safety was a facilitator for creating a culture of workplace safety. Most participants had limited knowledge of OSHA and their rights as workers, many expressing an interest in wanting to work in safer environments.

Our findings are similar to other studies conducted in community settings in which Spanish-speaking workers acknowledged working in hazardous conditions, often out of necessity (23–25). Similarly, the employer’s commitment to safety was crucial yet the incorporation of safety procedures was highly inconsistent (24, 25). A study by Roelofs et al. explored perceptions of OSHA in more detail, noting that workers had varying opinions about the actual influence of OSHA standards on safety (24). This is important considering existing OSHA policies related to ‘workers rights’ clearly define the rights and protections of employees, requiring employers to maintain a workplace that is free of hazards.^1^ Our findings suggest that OSHA regulations are not appropriately followed, leaving immigrant workers particularly vulnerable to injury. One minor theme that was noted in our study and reflected in others is the responsibility workers take for their own safety, recognizing employer limitations and the need and desire to protect themselves (23). Our findings combined with others suggest that employers can play a critical role in creating a culture of safety. However, we recognize that employers have varying levels of interest and incentives to do this and OSHA requirements appear to be poorly enforced.

Immigrant workers experience different layers of structural vulnerabilities, suggesting that perhaps immigration status *is* itself a risk factor for injury. The extraordinary unsafe occupational conditions Spanish-speaking workers face are not new and occupational disparities are well documented among this population (4–6). National organizations such as the Centers for Disease Control and Prevention (CDC) and National Institute for Occupational Safety and Health (NIOSH) have called for more research and tailored interventions specific to immigrant workers and several interventions have been documented. For example, a structured safety curriculum and mobile based video in Spanish with dairy workers at their work site, dissemination of safety posters, brochures and videos at the Mexican consulate, and a short educational video intervention in the community (19, 20, 26, 27). Multi-sector and multi-faceted strategies may be a key component in ensuring interventions address all levels, from individual to structural interventions; a true public health approach. To our knowledge, no hospital-based occupational injury prevention interventions for immigrant populations have been described.

Hospitals and health systems are increasingly recognized as having an important role in addressing social determinants of health and upstream factors that influence health (28). Hospital based injury prevention programs have been efficacious at reducing injury and violence *at* the point of care (29, 30). Implementing hospital based occupational injury prevention programs may be another important tool in addressing workplace injury. As noted at this study site, Spanish-speaking individuals routinely present to our hospital for work related injuries yet no culturally or linguistically tailored injury prevention intervention exists, leaving individuals who can return to work exposed to the same unsafe conditions that caused their injury. In addition to injury prevention strategies, it may be important to incorporate KYR training into a hospital based injury prevention toolkit given that participants had limited knowledge of their rights as workers. While KYR trainings have not been widely integrated in health settings, they are a routinely used strategy by civil liberties organizations (31, 32). This may be an important area for future research as few existing workplace interventions have incorporated KYR information. If individuals are unaware of their rights as workers, it may be difficult for them to advocate for safer work environments.

## Limitations

There are several limitations of our study. Interviews were conducted at a single hospital and thus may not be generalizable to other hospitals. Nonetheless, the hospital is the only Level 1 trauma and safety-net hospital, with a diverse patient population. Participants included were those who suffered a severe injury requiring hospitalization and themes may not necessarily reflect the experiences of those with injuries not requiring hospitalization or those who have not suffered an injury; however, the participants discussed previous injuries for which medical care was not sought. Interviews were conducted during the acute and/or subacute phase of the injury and may not capture the long term emotional and physical impacts of the injury. Most patients admitted to the hospital during this time period for work related injuries identified as men and only one woman was included in this study. Over half of immigrant women in the United States participate in the labor force, most commonly working in healthcare, hospitality, and food service. Immigrant women are also exposed to hazardous conditions, often work in informal sectors with woefully inadequate safety procedures or protection, and experience injury types that are different than men (33–35). The experiences of immigrant women are relatively understudied thus it is important that future studies explore the intersectionality of different identities (gender, race, ethnicity, immigration status) to better understand the experiences of all immigrant workers. Additionally, our study focused on traumatic work related injuries and did not explore non-injury related conditions. Previous studies have demonstrated that immigrant workers experience other occupational risks and occupational diseases with infectious diseases and metabolic cardiovascular disease being the most common (36). We did not include injured workers who spoke languages other than Spanish, who may face similar and unique challenges. Future studies should explore the experiences of diverse populations to better develop culturally and linguistically appropriate injury prevention and KYR resources. Finally, interviews were conducted in Spanish, transcribed, and translated into English thereafter. Coding and thematic analysis were performed utilizing English transcripts. As a result, it is possible that some words or concepts did not directly translate into English and therefore may have been unintentionally mistranslated and not incorporated into the analysis. However, this is unlikely as a professional transcription and translation service was used and coders were bilingual.

## Conclusion

The role of Immigration as a Social Determinant of Health is important to consider when identifying injury and violence-related public health threats. Often, an individual’s immigration status can dictate their options for *and* access to employment. The occupations that may be accessible to immigrants are high risk, hazardous jobs, where injuries are common. Indeed, based on our findings, immigrant workers continue to normalize working in hazardous, injury prone conditions feeling that safety is their own personal responsibility, rather than their employers. Participants voiced how important it is for employers to have a culture of safety to protect workers and how that would add to their feeling of safety at the workplace. Yet, injury prevention mechanisms and protections are highly variable. The inconsistent safety training and safety procedures at work places suggest that OSHA and agencies have a bigger role to play in protecting workers. Moreover, it suggests that public health approaches to injury prevention must account for this, recognizing that immigration status alone is a risk factor for injury and thus a threat to public health. The first step when considering a public health approach to injury prevention is to define the problem and then identify risk and protective factors. In this study, we suggest that immigration, an important social determinant of health, may be a risk factor for injury requiring a public health approach to address the problem. Hospital settings are an important location for injury prevention strategies yet few hospital-based injury prevention interventions specific to immigrant populations exist. Future studies are needed to develop and test hospital-based injury prevention strategies that protect workers from injury. As few workers are aware of their rights, interventions that incorporate rights based education, particularly for high-risk jobs, will be an important component to empower and protect workers. Implementing hospital based occupational injury prevention programs may be another important public health tool in addressing workplace injury and requires further research.

## Data availability statement

The raw data supporting the conclusions of this article will be made available by the authors, without undue reservation.

## Ethics statement

The studies involving humans were approved by Emory Institutional Review Board. The studies were conducted in accordance with the local legislation and institutional requirements. The participants provided their written informed consent to participate in this study.

## Author contributions

AZ: Conceptualization, Data curation, Formal analysis, Funding acquisition, Investigation, Methodology, Project administration, Resources, Software, Supervision, Validation, Visualization, Writing – original draft, Writing – review & editing. JC: Conceptualization, Data curation, Formal analysis, Investigation, Software, Writing – review & editing. HM: Formal analysis, Investigation, Software, Writing – review & editing. RC: Conceptualization, Formal analysis, Funding acquisition, Methodology, Resources, Supervision, Writing – review & editing. RS: Conceptualization, Formal analysis, Funding acquisition, Investigation, Resources, Supervision, Writing – review & editing. AS: Conceptualization, Formal analysis, Funding acquisition, Investigation, Project administration, Resources, Writing – review & editing. EZ: Conceptualization, Formal analysis, Funding acquisition, Investigation, Resources, Writing – review & editing. SA: Conceptualization, Data curation, Formal analysis, Funding acquisition, Investigation, Methodology, Project administration, Resources, Supervision, Visualization, Writing – review & editing.

## Footnotes

^1^https://www.osha.gov/sites/default/files/publications/osha3021.pdf
