# Supplementary figures and images for: “Caminando Con Riesgo”: perceptions of occupational injury, workplace safety and workers rights among Spanish-speaking hospitalized patients
Source: Front Public Health. 2024 Apr 23;12:1347534. doi: 10.3389/fpubh.2024.1347534 (PMC11074346; doi:10.3389/fpubh.2024.1347534)

**
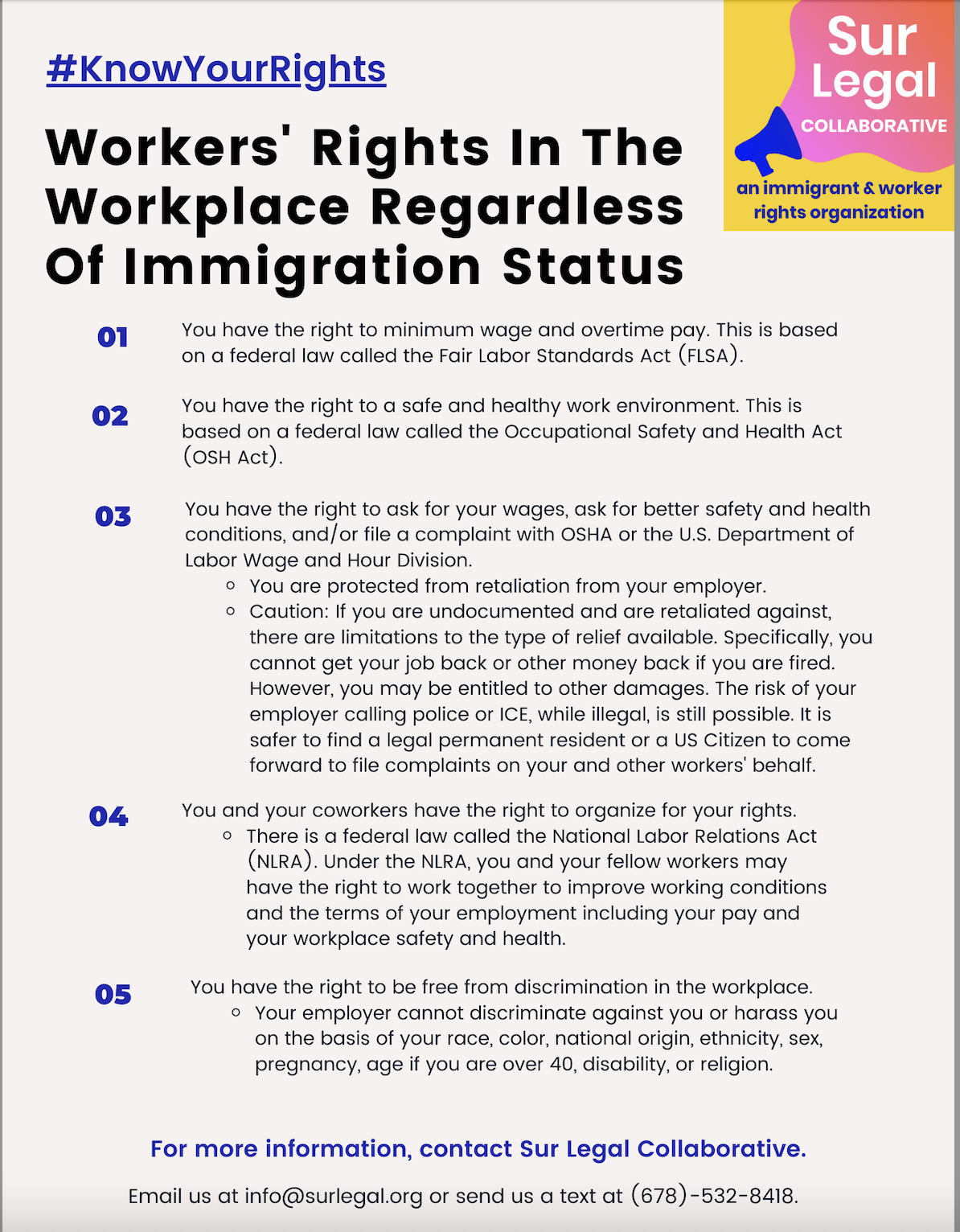
**

**
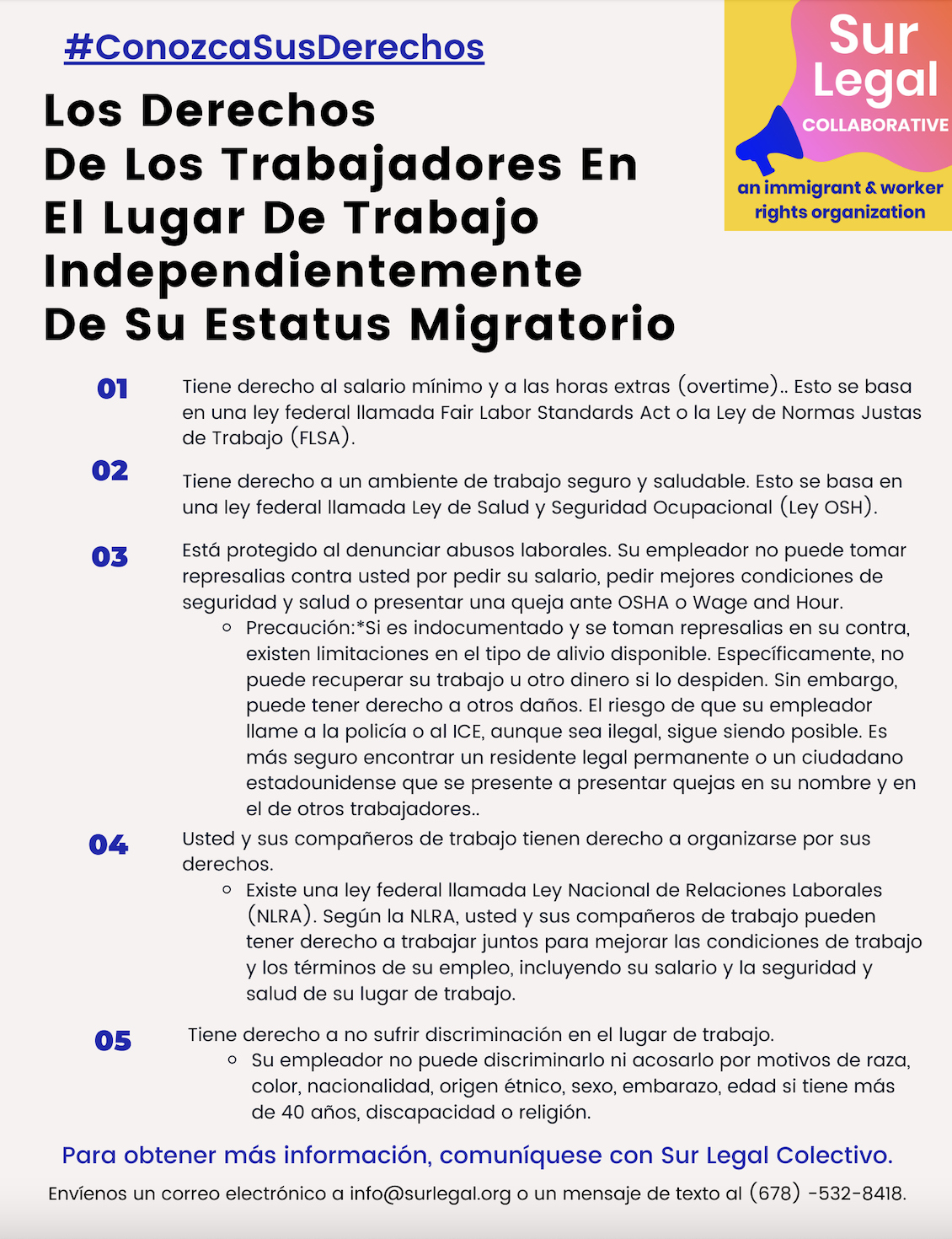
**

Supplement: Supplementary file 1 [file Data_Sheet_1.docx]
