# Supplementary material for: “Caminando Con Riesgo”: perceptions of occupational injury, workplace safety and workers rights among Spanish-speaking hospitalized patients
Source: Front Public Health. 2024 Apr 23;12:1347534. doi: 10.3389/fpubh.2024.1347534 (PMC11074346; doi:10.3389/fpubh.2024.1347534)
Supplement: Supplementary file 2 [file Data_Sheet_2.docx]

**Interview Guide**

As a reminder, during this interview, we will ask you about your work related injury, work conditions, and access to safety information at work.

We will be recording the interview to help us remember your responses. Is it okay with you if we record? We will not be recording your name or medical information.

I will start recording and we can begin the interview.

*Injury Related Questions*

First, I would like to ask you some questions about your work and how you were injured.

Can you tell me about what kind of work you do?

Probe: Can you describe what kind of activities you do at work?

Probe: Can you describe the environment you work in (outside, in a factory, driving,

etc.)

Is this the first time you have been injured at work? If no, can you describe other work related injuries you have experienced?

Can you tell me about how you were injured at work?

Probe: what kind of activity were you doing when you were injured?

Probe: were you using any type of equipment if injured, and if so can you describe it?

Probe: can you describe the environment where you were injured (outside, in a factory,

driving, etc.)

Have any of your co-workers been injured at work? What are the most common injuries you and your co-workers experience?

Why did you decide to come to the hospital for your injury?

Probe: Did anyone you work with encourage you to come to the hospital?

Probe: Did anyone you work with discourage you from coming to the hospital?

Being in the hospital for an injury is very stressful. Can you tell me how this injury has affected you personally?

How do you think this injury will affect your ability to work in the future?

Probe: Do you think you will return to the same job or similar type of work? why/why

Not?

*Work Safety Questions*

Next, I would like to ask you some questions about your safety while at work.

When you were injured at work, were you wearing any type of safety or protective equipment?

If yes, can you describe what you were wearing?

If no, why were you not wearing safety or protective equipment?

When you are at work, what safety measures do you use to protect yourself?

Probe: What kind of protective/safety equipment do you use?

Probe: Do you take breaks if working in extreme conditions (really hot, cold, loud, etc.)

Can you talk about some things your employer does to make sure your work environment is safe?

Probe: Does your employer provide safety equipment? If so, what kinds?

Probe: Has your employer talked to you about workplace safety? If so, what kind of

safety information and or education did they provide?

Do you feel safe at work?

If yes: Can you describe what makes you feel safe at work?

If no: Can you describe why you do not feel safe at work? describe why.

*Workers Rights Questions*

Finally, I would like to ask you some questions about your rights as a worker.

All employees have rights at work. Are you familiar with any of your rights at work (for example, your right to a safe and healthy work environment)?

Probe: Can you describe some of your rights as a worker?

There is an organization called the Occupational Safety and Health Administration (OSHA). This organization makes sure that all workers have a safe and healthy work environment. Have you ever heard of this organization before?

If yes: can you tell me how you learned about this organization?

Do you think your employer follows OSHA standards to make sure you are safe at work?

Probe: If not sure: Do you think your employer takes the necessary precautions to ensure you are safe at work?

What are some things your employer could do to make sure there is a safe working environment for workers?

What does your employer do that helps protect workers?

Do you know if your employer reported your current injury and hospitalization to OSHA?

What kind of information would be helpful to you about preventing injuries at work?

Probe: how would you like to access this information (via a website, printed brochure,

video)?

What kind of information would be helpful to you to better understand your rights as a worker?

Probe: how would you like to access this information (via a website, printed brochure,

video)?

Is there anything we haven’t talked about that you would like to add that may be important for this study?

Thank you so much for participating. I am going to stop the recording and ask you a few more questions that we will not be recording. You do not need to answer if you do not feel comfortable.

STOP RECORDING

**Study Demographics (Revised X/X/X)**

Gender

Race/Ethnicity

Age

Country of Birth

Years living in the US

Preferred language

Other languages spoken

Number of years in current job

Highest education level completed

Do you have health insurance (y/n/unsure)

Language interview was conducted in

Interpreter Used

If yes, language of interpretation
