# Supplementary material for: “Caminando Con Riesgo”: perceptions of occupational injury, workplace safety and workers rights among Spanish-speaking hospitalized patients
Source: Front Public Health. 2024 Apr 23;12:1347534. doi: 10.3389/fpubh.2024.1347534 (PMC11074346; doi:10.3389/fpubh.2024.1347534)
Supplement: Supplementary file 3 [file Data_Sheet_3.docx]

**Codebook**

| Code name | Definition |
| --- | --- |
| Work setting | Where the participant primarily works -outside, inside, factory, commercial, residential, alone, etc. |
| Types of equipment used at work | Any equipment that is used on the job |
| First Injury | Whether the current encounter was their first injury and context around the injury |
| Reinjury | Whether the current encounter was a reinjury and context around reinjury |
| Coworker Injury | If a co-worker has been injured at the same job previously |
| Decision to seek care | Who made the decision to seek care, if employer or co-worker encouraged patient to see care. |
| Co-worker support | Co-worker relationships, support system, and employer support towards the individual employee/ employees. |
| Perceptions of employer/boss | General perceptions of employer, whether employer helps employee feel safe during work or does not; awareness of support during work day |
| Time from injury to presentation | Immediate vs delayed presentation to care; include text if patient presented elsewhere prior to presenting to this hospital |
| Awareness of injury reports | Any experience with individual awareness of previous injuries on the job site, knowledge of prior OSHA reports |
| Perception of Recovery | Participants' views on recovery and the extent of their injuries. |
| Concerns and consequences for returning to work | Participants can express either concerns of returning, not returning to work, or no concern for returning to work. |
| Environmental safety | Conditions in the environment that affect patient safety – include training/education (or lackthereof), include work temperature as well (very hot or very cold) |
| Awareness/knowledge of OSHA and OSHA policies, safety precautions, and reporting | Individuals' awareness of the Occupational Safety and Health Association regarding what it stands for and in what ways the organization serves the individual in their workplace, regulations, safety precautions |
| Safety equipment & measures | What safety equipment is often used for specific types of jobs..(can be different depending on job role), reporting of individuals' usage of their safety equipment at work or the assignment of equipment on job sites. Measures taken to maintain safety (ex breaks).  Also includes who supplies safety equipment – patient or employer |
| Feelings of safety | Individuals reporting of safety on the job site and in the work area that is conducted daily |
| Injury Type Mechanism | How they were injured, factors around injury including mechanism and injury. |
| Dangerous Conditions | Acknowledgement of risk on the job, regarding their tasks or their presence in a dangerous situation. |
| Emotions/impact related to Injury | Patients can express their emotions on how they personally feel about being injured or in the hospital. |
| Injury occurrence | If the patient was conducting an activity or were they stationary at a working site.  Describing the injury happening or situation/scenario leading to injury. |
| Information on Injury prevention | How patients would like to receive information on workplace safety and injury prevention |
| Awareness of and Knowledge of workers rights | If the participant is aware of their workplace labor rights, include text about required work breaks, vacation/sick days |
| Employee training | If participant expresses that they received **training** about their job and the type/description OR that there was a lack of training for the role prior to their injury. Includes training on injury prevention and safety measures. |
| Blame for injury | If participant comments on the fault of the injury - self, employer, co-workers |
| Suggested improvements related to OSHA, workers rights, safety generally | Any text in which the participants make a suggestion for how to improve safety, knowledge/access to workers rights information. |
