# Supplementary material for: “Caminando Con Riesgo”: perceptions of occupational injury, workplace safety and workers rights among Spanish-speaking hospitalized patients
Source: Front Public Health. 2024 Apr 23;12:1347534. doi: 10.3389/fpubh.2024.1347534 (PMC11074346; doi:10.3389/fpubh.2024.1347534)
Supplement: Supplementary file 4 [file Data_Sheet_4.docx]

**Appendix 4: COREQ (COnsolidated criteria for REporting Qualitative research) Checklist**

| **Topic & Number** | **Location** | **Question and Response** |
| --- | --- | --- |
| Domain 1: Research Team & Reflexivity |  |  |
| *Personal Characteristics* |  |  |
| 1. Interviewer | Methods | JC conducted all of the interviews |
| 2. Credentials | Methods | At the time of the study, JC was a public health student |
| 3. Occupation | Methods | At the time of the study, JC was a public health student |
| 4. Gender |  | All team members are women |
| 5. Experience and Training |  | The interviewer had qualitative experience with injured patients admitted to the trauma services at the study site. Interviews explored the nature of their injuries and experiences after discharge. The interviewer also had research experience as a research coordinator at the study site in the Department of Surgery. |
| *Relationship with Participants* |  |  |
| 6. Relationship established | Methods | There was no prior relationship with the participants and the interviewer. Although the interviewer was a research coordinator at the study site during the time of the interviews, participants were not enrolled in any other studies and therefore would have no interaction with the interviewer prior to the interview. The interviewer had no clinical experience and thus had no involvement in participant’s medical care. |
| 7. Participant knowledge of interviewer |  | Participants were told at the beginning of the interviewer that the interviewer was a research assistant. The interviewer read the consent form to each participant at the start of the interview, which included standardized text regarding the purpose and motivation of the interview:  *My name is Juliana and I am a research assistant with the department of emergency medicine and trauma surgery. We are conducting a survey study to understand work related injuries experienced by patients at Grady. Would you be interested in learning more about this study?*  *The purpose of this study is to understand work related injuries our patients experience. We would like to better understand what work related injuries you have experienced, your work conditions, and your access to safety information at work. This study will take about 30-45 minutes to complete. If you join, you will be asked to complete an interview with me. The interview includes questions about work related injuries you may be experiencing or have experienced in the past. We will not be asking for the name of your company or boss and this information will never be shared with your company or boss. Your name will not be associated with the responses.* |
| 8. Interviewer characteristics |  | The interviewer is a native Spanish speaker and because of her identity had an interest in the study topic. The motivations and interests of the interviewer were not explicitly told to the participants. While participants could have made assumptions about her identity, they were not directly told to participants. |
| **Domain 2: Study Design** |  |  |
| *Theoretical Framework* |  |  |
| 9. Methodological Orientation & Theory | Methods | We used a thematic analysis approach to analyze data as described by Braun and Clarke. This methodology offers a six-stage flexible approach to understand, describe and interpret often large amounts of complex data in order to develop insights about experiences centered around a particular question (e.g. contextual factors around work-related injuries requiring hospitalization, knowledge of OSHA and workers rights) |
| *Participant Selection* |  |  |
| 10. Sampling | Methods | Participants were sampled purposely to identify participants that were Spanish speaking. The study team reviewed demographics of participants at different points throughout the study to balance across occupation and injury type. |
| 11. Method of Approach | Methods | Participants were recruited in person at the study site hospital. The research assistant identified Spanish speaking patients admitted for a work related traumatic injury. Patients were approached in their private hospital rooms and interviews were conducted after consent was obtained. |
| 12. Sample Size | Results | A total of 8 participants were included in the study. |
| 13. Non-participation | Methods | No participants refused to participate or withdrew from the study. |
| *Setting* |  |  |
| 14. Setting of Data Collection | Methods | Interviews were conducted in person at the study site. Interviews took place in the patient’s private hospital room. |
| 15. Presence of non-participants |  | During the interview, no other individuals were present outside of the interviewer and participant. |
| 16. Description of Sample | Results | Participants self-identified as Latino or Hispanic and were from Mexico (n=3), Guatemala (n=3), Honduras (n=1) and El Salvador (n=1). The mean age was 39 with a range of 23 to 69 years old. The mean number of years living in the U.S. was 14 with a range of 3 to 30 years. All participants reported Spanish as their preferred language and two reported also speaking an indigenous language. They had worked in their current job for a range of 3 months to 8 years with a mean of 3.3 years. The highest level of education completed varied from 10 years of age (primary school) to one year of college with 14 years old being the mean age of highest education completed. Most did not have insurance (n=6).. |
| *Data Collection* |  |  |
| 17. Interview Guide | Methods | The interview guide was informed by the study team’s content expertise in occupational health, environmental health, nursing, public health, emergency medicine, trauma surgery and critical care, injury prevention, labor law, and workers rights, and their knowledge of evidence from existing literature. The interview guide was piloted and refined to verify that questions were appropriately understood by participants.  The interview guide is included in the appendix. |
| 18. Repeat Interviews |  | No repeat interviews were conducted. |
| 19. Audio/visual Recording | Methods | All interviews were recorded on a handheld recorder.. The audio recordings were saved via a secure password protected online storage system and uploaded to a secure HIPPA compliant transcription system. Spanish transcripts were translated into English using a professional translation service. English transcripts were reviewed, de-identified and saved via a secure password protected online storage system |
| 20. Field Notes |  | While a field note template was generated and available for interviews, the interviewer did not identify a need to utilize field notes. |
| 21. Duration | Results | Interviews lasted approximately 45-60 minutes. |
| 22. Data Saturation | Methods | Thematic saturation was reached. |
| 23. Transcripts Returned |  | Transcripts were not returned to participants for review. |
| **Domain 3: Analysis & Findings** |  |  |
| *Data Analysis* |  |  |
| 24. Number of Data Coders | Methods | After an initial review of transcripts, study team members trained in qualitative analysis (JC, HM, AJZ) developed a preliminary code book and open coded a sample of transcripts. The preliminary code book was refined and a final code book was developed. The initial transcripts coded using the preliminary code book were re-coded using the final code book. All remaining transcripts were coded by two separate coders (JC and HM) |
| 25. Description of the Coding Tree | Appendix | The coding tree is included in the appendix. Codes included: work setting, types of equipment used at work, first injury, reinjury, coworker injury, decision to seek care, co-worker support, perceptions of employer/boss, time from injury to presentation, awareness of injury reports, perception of recovery, concerns and consequences for returning to work, environmental safety, Awareness of OSHA, safety equipment and measures, feelings of safety, injury type mechanism, dangerous conditions, emotions related to injury, injury occurrence, information on injury prevention, awareness of workers rights, employee training, blame for injury, suggested improvements. |
| 26. Derivation of Themes | Methods | Themes were derived from the data. |
| 27. Software | Methods | Dedoose was used for coding. |
| 28. Participant Checking |  | Participants did not provide feedback on the findings. |
| 29. Quotations Presented | Table 1 | Quotations were included in Table 1 to represent prominent themes. |
| 30. Data & Findings Consistent | Results | There is consistency between the data presented and the findings. |
| 31. Clarity of Major Themes | Results | Major themes are presented in the results section. |
| 32. Clarity of Minor themes | Results | Minor themes are presented in the results section. |
